# Supplementary material for: Genome-Wide Identification of Mitochondrial Calcium Uniporter Family Genes in the Tomato Genus and Expression Profilings Under Salt Stress
Source: Curr Issues Mol Biol. 2025 Dec 7;47(12):1021. doi: 10.3390/cimb47121021 (PMC12732091; doi:10.3390/cimb47121021)
Supplement: Supplementary file 1 [file cimb-47-01021-s001.zip › cimb-4009822-supplementary.pdf]

Supplementary Table S1. Physicochemical properties of wild tomato MCU proteins.

	Protein identity	Chromosome location	Amino acids	Theoretical pI	Molecular weight	Instability index	Aliphatic index	GRAVY	Protein secondary structure			Subcellular Localization
									Alpha helix	Beta turn	Random coil	
S.chilense	*SchiMCU1*	chr2	349	9.03	39714.28	55.69	86.02	-0.215	176	9	138	mito
	SchiMCU2	chr2	354	8.97	40640.1	48.43	71.95	-0.328	177	10	138	mito
	SchiMCU3	chr3	303	9.27	35848.98	38.99	73.66	-0.356	161	10	111	mito
	SchiMCU4	chr4	332	9.39	38666.11	52.32	90.42	-0.232	175	9	119	chlo
	SchiMCU5	chr6	304	9.09	34783.41	35.36	94.84	-0.223	157	22	80	chlo_mito
	SchiMCU6	chr6	304	9.1	34771.4	35.92	95.16	-0.231	162	19	84	mito
S.chmielewskii	*SchmMCU1*	chr2	93	8.84	11387.23	60.17	57.63	-0.303	49	4	23	chlo
	SchmMCU2	chr2	354	8.74	40657.04	50.73	71.95	-0.335	181	8	134	nucl
	SchmMCU3	chr3	319	9.43	38129.64	41.81	75.14	-0.356	177	9	107	mito
	SchmMCU4	chr3	321	9.35	37438.59	45.88	85.36	-0.294	181	8	102	mito
	SchmMCU5	chr4	332	9.39	38620.08	50.36	90.42	-0.22	167	8	130	chlo
	SchmMCU6	chr6	304	9.1	34825.45	36.55	95.16	-0.226	178	13	71	chlo_mito
S.corneliomulleri	*ScorMCU1*	chr2	100	8.82	12145.24	56.4	72.1	-0.055	50	3	27	chlo
	ScorMCU2	chr2	354	9.01	40588.08	50.75	72.49	-0.326	175	12	137	nucl
	ScorMCU3	chr3	303	9.5	35881.08	41.54	76.86	-0.364	167	11	103	mito
	ScorMCU4	chr3	321	9.66	37592.79	49.34	82.62	-0.349	173	8	112	mito
	ScorMCU5	chr4	332	9.45	38562.04	51.31	90.72	-0.205	174	10	124	chlo
	ScorMCU6	chr6	304	9.1	34757.38	34.58	94.84	-0.238	161	21	81	mito
S.lycopersicum _var_ cerasiforme	*SlyvMCU1*	chr2	103	9.2	12441.57	60.74	66.21	-0.096	56	3	25	chlo
	SlyvMCU2	chr2	354	9.14	40573.08	50.83	74.15	-0.319	173	10	140	mito
	SlyvMCU3	chr3	301	9.4	35720.84	40.11	74.78	-0.369	171	10	105	mito
	SlyvMCU4	chr3	321	9.42	37588.74	44.93	83.52	-0.336	172	11	117	mito
	SlyvMCU5	chr4	332	9.45	38677.13	52.15	89.55	-0.247	173	10	126	chlo
	SlyvMCU6	chr6	304	9.1	34771.4	35.92	95.16	-0.231	162	19	84	mito
S.galapagense	*SgalMCU1*	chr2	109	9.56	13345.76	51.95	69.72	-0.114	48	1	34	cyto
	SgalMCU2	chr2	354	8.97	40574.02	50.4	74.15	-0.318	179	9	135	mito

	SgalMCU3	chr3	291	9.43	34608.44	36.49	71.99	-0.403	168	11	92	mito
	SgalMCU4	chr3	321	9.37	37576.75	42.36	82.9	-0.327	169	11	115	mito
	SgalMCU5	chr4	332	9.45	38677.13	52.15	89.55	-0.247	173	10	126	chlo
	SgalMCU6	chr6	304	9.1	34771.4	35.92	95.16	-0.231	162	19	84	mito
S.habrochaites	*ShabMCU1*	chr2	342	8.94	39060.36	54.69	83.22	-0.285	180	12	124	mito
	ShabMCU2	chr2	354	8.86	40520.93	47.67	73.05	-0.315	172	11	137	nucl
	ShabMCU3	chr3	313	9.55	37008.49	41.47	75.05	-0.379	166	11	116	mito
	ShabMCU4	chr3	321	9.51	37620.8	46.76	87.45	-0.309	178	9	115	mito
	ShabMCU5	chr4	332	9.45	38693.13	52.77	89.25	-0.249	172	8	128	chlo
	ShabMCU6	chr6	304	9.09	34797.44	35.36	95.16	-0.224	174	14	77	chlo_mito
S.lycopersicoides	*SlydMCU1*	chr2	92	9.12	11195.02	61.16	58.26	-0.293	45	5	22	chlo
	SlydMCU2	chr2	169	8.67	19713.04	41.01	57.22	-0.312	105	4	46	mito
	SlydMCU3	chr4	332	9.56	38622.96	53.02	88.07	-0.249	177	9	123	chlo
	SlydMCU4	chr6	312	9	35416.9	32.27	91.79	-0.281	159	19	91	mito
S.neorickii	*SneoMCU1*	chr2	93	8.84	11387.23	60.17	57.63	-0.303	49	4	23	chlo
	SneoMCU2	chr2	354	8.81	40520.97	49.85	74.15	-0.298	177	9	137	mito
	SneoMCU3	chr3	303	9.04	35884.9	39.65	74.29	-0.338	169	9	105	mito
	SneoMCU4	chr3	321	9.49	37666.85	43.93	83.83	-0.312	179	9	110	mito
	SneoMCU5	chr4	332	9.4	38646.03	53.29	89.25	-0.26	183	8	116	chlo
	SneoMCU6	chr6	304	9.09	34797.44	35.36	95.16	-0.224	147	14	77	chlo_mito
S.pennellii	*SpenMCU1*	chr2	349	9.08	39846.19	53.92	78.19	-0.32	197	9	124	chlo
	SpenMCU2	chr2	323	6.97	37045.8	49.55	70.99	-0.36	167	9	120	nucl
	SpenMCU3	chr3	321	9.6	37581.77	47.6	84.14	-0.313	177	10	117	mito
	SpenMCU4	chr4	332	9.45	38653.07	53.07	89.25	-0.245	171	8	129	chlo
	SpenMCU5	chr6	304	9.18	34830.53	36.88	94.21	-0.249	161	21	79	mito
S.peruvianum	*SperMCU1*	chr2	349	9.08	39774.15	56.38	82.38	-0.302	179	10	137	chlo_mito
	SperMCU2	chr2	354	8.92	40550.03	50.94	72.77	-0.328	174	8	145	mito
	SperMCU3	chr4	332	9.45	38728.23	51.81	90.12	-0.231	169	8	132	chlo
	SperMCU4	chr6	304	9.1	34757.38	34.58	94.84	-0.238	161	21	81	mito

	SpimMCU1	chr2	349	9.08	39759.18	56.38	82.95	-0.288	187	9	133	chlo_mito
	SpimMCU2	chr2	354	8.97	40574.02	50.4	74.15	-0.318	179	9	135	mito
	SpimMCU3	chr3	303	9.34	35949.08	39.92	75.25	-0.364	168	10	105	mito
S.pimpinellifolium	*SpimMCU4*	chr3	321	9.42	37588.74	44.93	83.52	-0.336	172	11	117	mito
	SpimMCU5	chr4	332	9.45	38677.13	52.15	89.55	-0.247	173	10	126	chlo
	SpimMCU6	chr6	304	9.1	34771.4	35.92	95.16	-0.231	162	19	84	mito

[1] Grand Average of Hydropathicity (GRAVY), Positive values indicate that the protein is hydrophobic, and negative values indicate that the protein is hydrophilic.

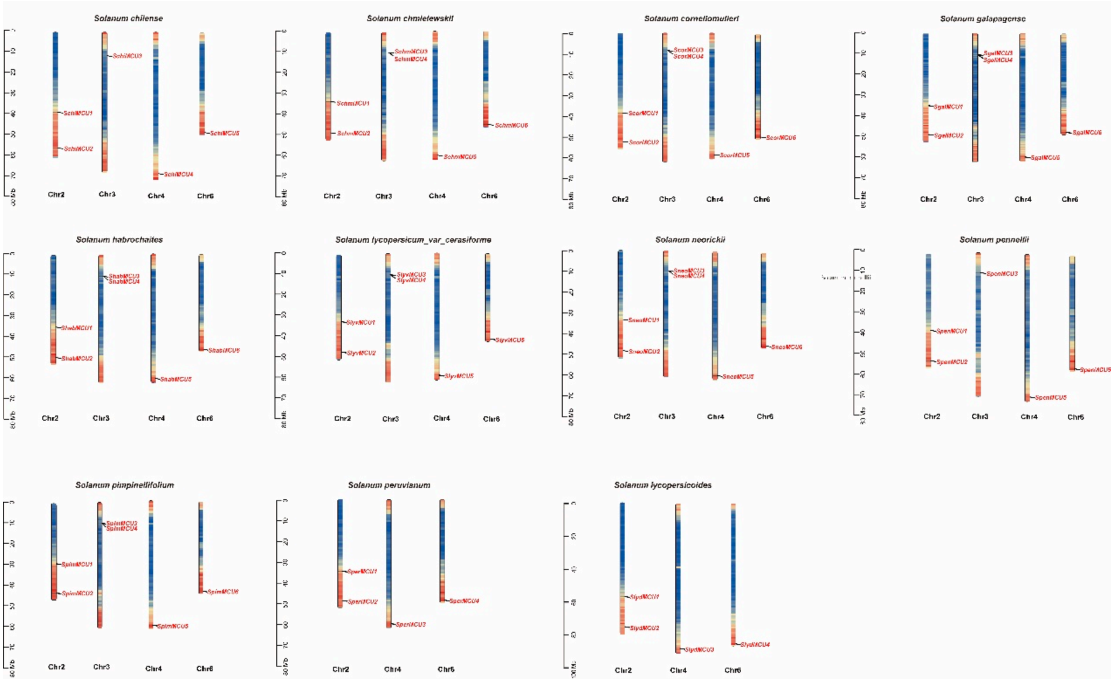

Supplementary Figure S1. Chromosomal localization of the wild tomato MCU genes.
